# Causal association between iron status and heart failure: A 2-sample bidirectional Mendelian randomization study

**DOI:** 10.1097/MD.0000000000045391

**Published:** 2025-11-07

**Authors:** Yaqi Hu, Tao He, Jianhe Liu, Weisong Wang

**Affiliations:** a National Chinese Medicine Master Sun Guangrong Studio, Hunan Academy of Chinese Medicine, Changsha, Hunan, China; b Department of Cardiology, The First Hospital of Hunan University of Chinese Medicine, Changsha, Hunan, China.

**Keywords:** causal inference, ferritin, heart failure, iron, Mendelian randomization

## Abstract

Observational studies have shown that serum iron status is related to heart failure (HF). However, due to confounding factors and reverse causation, there is still controversy over whether this association is causal. Our aim is to assess the causal relationship between genetically predicted serum iron status and HF. This study uses inverse variance weighting as the main method for Mendelian Randomization (MR) analysis, while also employing MR-Egger and weighted median as supplementary methods to evaluate the causal relationship between exposure factors and outcome variables. Additionally, the MR-Egger intercept test and MR-pleiotropy residual sum and outliers (MR-PRESSO) test were employed to assess the horizontal pleiotropy and outlier single nucleotide polymorphism. Cochran Q test in MR-Egger and inverse variance weighting methods was performed to evaluate the heterogeneity among the single nucleotide polymorphisms, and sensitivity analysis was performed by using the“leave-one-out”method. Finally, reverse MR analysis was used to verify the robustness of the results. Elevated serum ferritin levels (OR = 1.147, 95% CI 1.014–1.298, *P*-value = .029) have a causal relationship with a reduced risk of HF. However, there is no potential genetic causal relationship between serum iron, total iron-binding capacity, and transferrin saturation and HF (*P* >.05). In the reverse MR analysis, none of the 3 methods support reverse causality. The intercept test of the MR-Egger regression model, the MR-PRESSO test, and Cochran Q test all have *P*-values >.05, indicating that the selected single nucleotide polymorphisms do not exhibit horizontal pleiotropy or heterogeneity. In addition, the sensitivity analysis based on the leave-one-out method shows that a single single nucleotide polymorphism does not affect the robustness of the causal association effect value. There is a causal relationship between genetically predicted serum ferritin levels and the risk of HF, indicating that high levels of serum ferritin may be a protective factor against HF.

## 1. Introduction

Heart failure (HF) is a severe and terminal stage of various organic heart diseases, characterized by the heart’s inability to supply the necessary blood and oxygen to peripheral tissues to meet their metabolic needs. It is one of the most critical cardiovascular diseases of the 21st century.^[1]^ Its pathological mechanisms are complex, involving cardiomyocyte apoptosis, energy metabolism disorders, immune inflammatory responses, and ferroptosis.^[2,3]^ In recent years, with the changes in the concept and technology of HF treatment, the mortality rate has decreased somewhat, but the overall mortality rate remains high.^[1]^ The fundamental reason for this is the limited understanding of the pathogenesis of HF. Exploring the pathogenesis of diseases through causal relationships is crucial for taking targeted intervention measures and preventing the occurrence of diseases.

Iron is an essential nutrient required for many biological processes. Both extremes of iron status are associated with adverse cardiovascular outcomes.^[4]^ Patients with HF exhibit a disturbance in iron metabolism, characterized by both circulatory iron deficiency and iron overload in myocardial tissue. Circulatory iron deficiency leads to anemia and diminished exercise capacity; from this perspective, supplementing with iron can help alleviate patients’ symptoms.^[5]^ Excess iron in cardiomyocytes has been shown to induce ferroptosis, a type of programmed cell death driven by iron-dependent lipid peroxidation, associated with cardiovascular diseases.^[6]^ Research has confirmed that mitochondrial iron overload may be involved in the pathophysiological process of HF, and the use of mitochondrial iron chelators may be effective in delaying the progression of HF.^[7]^ The research conclusions on the relationship between iron status and HF are inconsistent.^[8,9]^ Therefore, the causal relationship between iron status and HF is still unclear, and it is necessary to conduct more in-depth studies on their correlation at the genetic level.

Mendelian randomization(MR) is a method based on summary data from large-sample genome-wide association studies (GWAS). It uses single nucleotide polymorphisms (SNPs) that areclosely related to exposure factors as instrumental variables (IV) to infer the causal relationship between exposure factors and outcome variables, and it is widely used to infer the causal relationships between various phenotypes and diseases.^[10]^ However, there is currently a lack of studiesusing MR methods to explore the relationship between iron status and HF. In this regard, this study employs a 2-sample bidirectional MR approach to analyze the causal relationships between 4 indicators related to iron status: serum iron, ferritin, total iron-binding capacity (TIBC), transferrin saturation (TSAT), and HF.

## 2. Methods

### 2.1. Study design

This study is designed as a 2-sample bidirectional MR analysis. First, 4 iron status-related indicators are treated as exposure factors, and HF is treated as the outcome variable for MR analysis. Secondly, HF is treated as the exposure factor, and iron status-related indicators are treated as the outcome variables for reverse MR analysis. The MR analysis in this study follows 3 key assumptions in order to effectively control for potential confounding factors and reverse causation, thereby ensuring reliable estimates of causal effects.^[11]^ Association assumption: SNPs used as IV must beclosely related to the exposure factors; Independence assumption: SNPs used as IV must be independent of any otherconfounding factors; Exclusion assumption: The effect of the IV on the outcome variable can only occur through the exposure factors, and not through other means.

### 2.2. Data sources

The summary data of iron status-related indicators comes from a MR study published by Alexa et al in 2024. The summary data is derived from the largest Fe GWAS to date, which includes a meta-analysis of GWAS from 6 European populations: DeCODE, INTERNAL, SardiNIA, Danish Blood Donor Study, Trøndelag Health Study (HUNT), and Michigan Genomics Initiative.^[4]^ These indicators are serum ferritin, serum iron, TIBC, and TSAT.

The Heart Failure Molecular Epidemiology Treatment Targets (HERMES) Alliance. The HERMES Alliance has a large-sample size, including 47,309 cases from 17 population cohorts (38,780 cases) and 9 case-control samples (8529 cases) as well as 930,014 European ancestry controls.^[12]^ Detailed information are shown in Table 1.

**Table 1:** Details of data sources included in the study.

	Data source	Population	Sample size/cases/controls	Sex
Iron status				
TSAT	HUNT, MGI, DeCODE, INTERVAL	European	198,516	M/F
Serum Iron	HUNT, MGI, DeCODE, INTERVAL, SardiNIA	European	236,612	M/F
TIBC	HUNT, MGI, DeCODE, INTERVAL, SardiNIA	European	208,422	M/F
Ferritin	HUNT, MGI, DeCODE, INTERVAL, DBDS	European	257,953	M/F
Disease				
HF	HERMES	European	47,309	M/F

DBDS = Danish Blood Donor Study, HERMES = heart failure molecular epidemiology treatment targets, HF = heart failure, HUNT = Trøndelag health study, MGI = Michigan genomics initiative, TSAT = transferrin saturation, TIBC = total iron-binding capacity.

### 2.3. Instrumental variable selection

In the forward MR analysis, the IV are screened according to the following criteria: Extract SNPs that are strongly correlated with exposure factors such as serum iron, ferritin, TIBC, and TSAT from the GWAS summary data related to iron status indicators (*P* < 5 × 10^−8^). Select SNPs with a linkage disequilibrium coefficient *r*^2^ <0.001 and a clump distance >10,000 kb as candidate IV.^[13]^ Use the LDtrait database (https://ldlink.nih.gov/tab=home) to conduct confounding analysis on SNPs, assessing whether the SNPs may be related to confounding factors or outcome risk factors. SNPs corresponding to phenotypes related to the outcome are excluded, and the remaining SNPs are used as instrumental variables (IV) for clear causal inference. Specifically, when HF is the outcome, we exclude SNPs associated with confounding factors related to other cardiovascular diseases such as coronary atherosclerotic heart disease, primary hypertension, atrial fibrillation, type 2 diabetes, dyslipidemia, etc. Extract SNPs associated with serum iron, ferritin, TIBC, and TSAT from the GWAS summary data on the outcome variable heart failure. Then, coordinate and merge the datasets of exposure factors and outcome variables using the effect allele frequency, sequentially excluding SNPs with palindromic structures and those directly related to the outcome variable (*P* < 5 × 10^−8^). Calculate the F value to estimate the strength of the IV, and select SNPs with *F* >10 as the final IV.^[13]^ In the reverse MR analysis, independent SNPs significantly associated with heart failure (*P* < 5 × 10^−8^) were selected from GWAS summary data as IV. Other selection criteria were the same as those in the forward MR analysis. Similarly, in the reverse MR analysis, we excluded SNPs associated with iron status (serum iron, ferritin, TIBC, and TSAT).

### 2.4. Statistical analysis

This study uses inverse variance weighting (IVW) as the primary method, while also employing MR-Egger and weighted median (WM) regression models as supplementary methods for MR analysis. It interprets the causal relationship between exposure factors and outcome variables using odds ratios (OR) and 95% confidence intervals (95% CI).^[14]^ Using the intercept term from MR-Egger regression and MR-pleiotropy residual sum and outlier (MR-PRESSO) to assess the horizontal pleiotropy of SNPs and identify outlier SNPs, it can be concluded that there is no horizontal pleiotropy when the intercept term is statistically nonsignificant compared to 0 (*P* >.05). At the same time, the MR-pleiotropy MR-PRESSO method is used to test for outlier SNPs and remove these abnormal SNPs to reduce the impact of horizontal pleiotropy on the causal effect estimates. Using Cochran *Q* test in IVW and MR-Egger regression to assess the heterogeneity of the IV, a *P*-value >.05 indicates no heterogeneity. Sensitivity analysis used the leave-one-out method, sequentially removing each SNP and then calculating the causal effect estimates of the remaining SNPs. If the overall results do not change significantly, it can be considered that there is no main effect SNP that has a large impact on the causal effect estimates. The above statistical analysis was completed using R software (version 4.4.2), TwoSampleMR (version 0.68), and MR-PRESSO (version 1.0) packages.

## 3. Results

### 3.1. Tool variable selection situation

According to the screening criteria for IV, in the analysis exploring the causal relationship between iron status and heart failure, a total of 51 SNPs associated with serum ferritin, 21 SNPs associated with serum iron, 38 SNPs associated with TIBC, and 21 SNPs associated with TSAT were included. When studying the impact of heart failure on iron status, 9 SNPs that met the screening criteria were found for each of the 4 outcome phenotypes: serum iron, ferritin, TIBC, and TSAT. The F values of all the aforementioned SNPs were >10, indicating that the possibility of weak instrumental variable bias is relatively low.

### 3.2. The effect of iron status on HF

**Figure 1.**
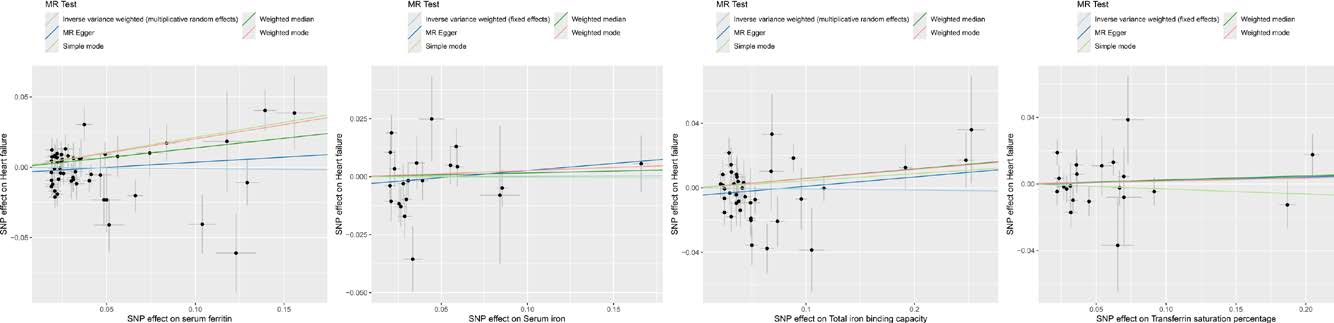
Scatter plot of the relationship between iron status and HF. HF = heart failure.

Using serum iron, ferritin, TIBC, and TSAT as exposure factors, and heart failure as the outcome variable, MR analysis was conducted. The results from the WM method indicate that a genetically predicted increase in serum ferritin levels (OR = 1.147, 95% CI 1.014–1.298, *P* = .029) is associated with a lower risk of heart failure. Although the results of the MR-Egger regression and IVW tests were not statistically significant (*P* >.05), the OR values and scatter plots indicate that these 2 methods showed similar trends to the WM method. Furthermore, MR-Egger regression, WM, and IVW all showed no causal relationship between serum iron, TIBC, and TSAT and heart failure (*P* >.05) (Table 2 and Fig. 1).

**Table 2:** MR analysis results of the relationship between iron status and HF.

Exposure	MR method	nSNP	OR	95% CI	*P*-value
Ferritin	MR-Egger	51	1.076	0.911–1.271	.395
	WM	51	1.147	1.014–1.298	.029*
	IVW	51	0.99	0.903–1.086	.833
Serum Iron	MR-Egger	21	1.063	0.891–1.268	.504
	WM	21	1.016	0.908–1.138	.78
	IVW	21	1.001	0.924–1.086	.969
TIBC	MR-Egger	38	1.059	0.945–1.187	.32
	WM	38	1.06	0.975–1.152	.17
	IVW	38	0.993	0.925–1.066	.846
TSAT	MR-Egger	21	1.023	0.909–1.152	.707
	WM	21	1.024	0.930–1.130	.621
	IVW	21	1.019	0.954–1.088	.576

95% CI = 95% confidence intervals, HF = heart failure, IVW = inverse variance weighting, MR = Mendelian randomization, OR = odds ratios, SNP = single nucleotide polymorphism, TSAT = transferrin saturation, TIBC = total iron-binding capacity.

* *P* <.05.

**Figure 2.**
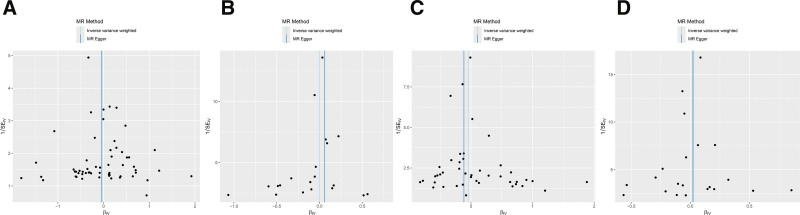
Funnel plot of MR analysis on the causal relationship between iron status and HF. (A) Ferritin. (B) Serum iron. (C) TIBC. (D) TSAT. HF = heart failure, MR = Mendelian randomization, TSAT = transferrin saturation, TIBC = total iron-binding capacity.

Using the MR-Egger regression model intercept test and MR-PRESSO test to assess the pleiotropic effects of serum iron, ferritin, TIBC, and TSAT on HF. The results showed that there is no horizontal pleiotropy (*P* >.05) between the SNPs of serum iron, ferritin, TIBC, and TSAT and the outcome of HF, indicating that the MR analysis in this study is an effective causal inference method (Table 3). In addition, according to the funnel plot, the distribution of all SNPs included in the analysis shows a shape that is basically symmetrical on both sides. This indicates that the use of these SNPs as genetic IV to infer causal relationships is less influenced by other potential confounding factors (Fig. 2).

**Table 3:** Horizontal pleiotropy and heterogeneity test.

Exposure	Horizontal pleiotropy				Heterogeneity			
	MR-Egger	*P*-value	MR-PRESSO Global	*P*-value	IVW Cochran Q	*P*-value	MR-Egger Cochran Q	*P*-value
Ferritin	0.002	.964	67.408	.235	64.673	.226	64.671	.2
Serum Iron	-0.003	.429	32.398	.082	30.676	.06	29.655	.056
TIBC	0.005	.34	51.546	.209	49.162	.208	48.07	.208
TSAT	-0.003	.927	27.835	.228	24.661	.215	24.65	.172

IVW = inverse variance weighting, MR = Mendelian randomization, MR-PRESSO = MR-pleiotropy residual sum and outliers, TSAT = transferrin saturation, TIBC = total iron-binding capacity.

**Figure 3.**
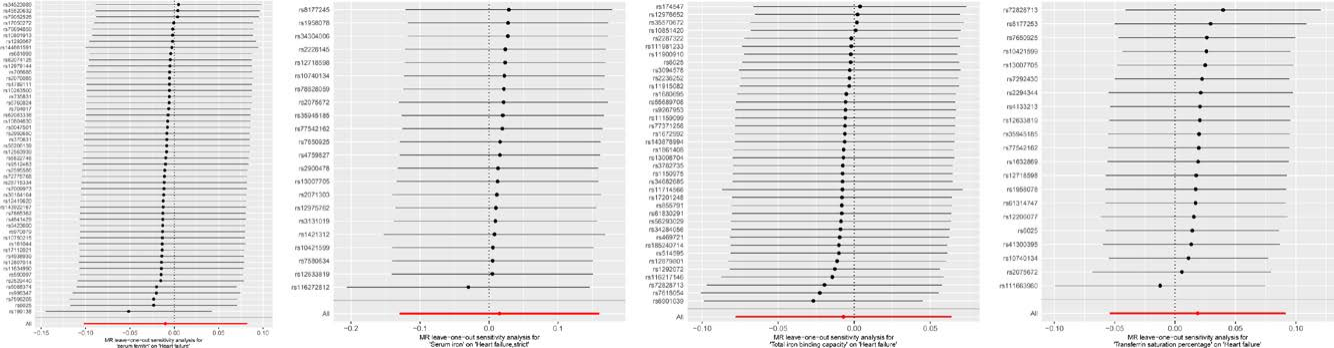
Leave-one-out sensitivity analysis of the causal relationship between iron status and HF. HF = heart failure.

Sensitivity analysis based on leave-one-out method assesses the impact of each SNP on the overall causal effect. The results show that removing a specific SNP from those associated with serum iron, ferritin, TIBC, and TSAT does not change the overall causal effect. This suggests that the causal effect is not driven by a single gene, and there is no principal effect SNP, which further supports the accuracy and stability of the MR analysis results (Fig. 3).

### 3.3. The impact of HF on iron status

The reverse MR analysis results, using heart failure as the exposure variable and iron status-related indicators as the outcome variables, show that the test results from the 3 methods are not statistically significant (*P* >.05). This indicates that there is no causal relationship between genetically predicted heart failure and serum iron, ferritin, TIBC, and TSAT (Table 4).

**Table 4:** MR analysis of the impact of HF on iron status.

Exposure	MR method	nSNP	OR	95% CL	*P*-value
Ferritin	MR-Egger	9	1.046	0.571–1.915	.889
	WM	9	1.045	0.984–1.109	.151
	IVW	9	0.971	0.809–1.165	.75
Serum Iron	MR-Egger	9	0.977	0.759–1.257	.86
	WM	9	0.992	0.935–1.052	.78
	IVW	9	0.968	0.897–1.044	.396
TIBC	MR-Egger	9	0.91	0.755–1.098	.357
	WM	9	1.059	0.992–1.130	.084
	IVW	9	1.068	0.998–1.143	.056
TSAT	MR-Egger	9	1.032	0.842–1.265	.768
	WM	9	0.966	0.905–1.030	.292
	IVW	9	0.941	0.897–0.986	.115

95% CI = 95% confidence intervals, HF = heart failure, IVW = inverse variance weighting, MR = Mendelian randomization, OR = odds ratios, SNP = single nucleotide polymorphism, TSAT = transferrin saturation, TIBC = total iron-binding capacity, WM = weighted median.

**Figure 4.**
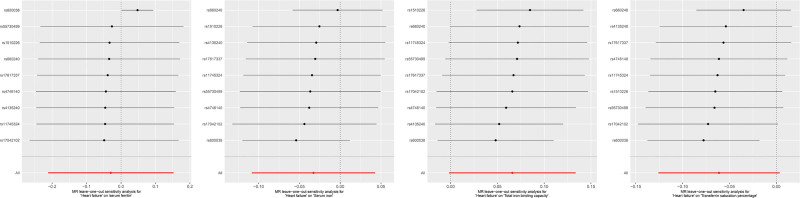
Sensitivity analysis of the reverse MR analysis results based on the leave-one-out method. MR = Mendelian randomization.

The *P*-values of the MR-Egger regression model intercept test, MR-PRESSO Global test, and Cochran *Q* test are all >.05, indicating that the selected SNPs do not exhibit horizontal pleiotropy and heterogeneity. Additionally, the sensitivity analysis based on the leave-one-out method shows that a single SNP does not affect the robustness of the causal association effect values (Table 5 and Fig. 4).

**Table 5:** Heterogeneity and pleiotropy of reverse MR analysis.

Outcome	Horizontal pleiotropy				Heterogeneity			
	MR-Egger	*P*-value	MR-PRESSO Global	*P*-value	IVW Cochran Q	*P*-value	MR-Egger Cochran Q	*P*-value
Ferritin	−0.005	.807	8.079	.527	137.921	6.419	136.661	2.543
Serum Iron	−0.006	.942	3.868	.818	24.866	.016	24.845	.008
TIBC	0.012	.12	19.159	.059	15.863	.444	0.957	.14
TSAT	−0.007	.376	18.786	.084	14.881	.061	13.12	.067

IVW = inverse variance weighting, MR = Mendelian randomization, MR-PRESSO = MR-pleiotropy residual sum and outliers, TSAT = transferrin saturation, TIBC = total iron-binding capacity.

## 4. Discussion

MR, as an analytical method that utilizes genetic variation as an instrumental variable, effectively overcomes the main limitations of observational studies, such as the influence of potential confounding factors and reverse causation on results. It provides a new and convenient way to infer causal relationships, similar to randomized controlled trials.^[14,15]^ Considering that previous studies on the relationship between iron status and HF have yielded inconsistent conclusions,^[16–19]^ this study uses a 2-sample bidirectional MR method for the first time to analyze the causal relationship between iron status and HF from the perspective of genetic variations. The results show that elevated serum ferritin levels predicted by genetics are causally related to a lower risk of HF. No potential genetic causal relationships were found between serum iron, TIBC, and TSAT and HF. In contrast, reverse MR analysis did not find a potential causal relationship between HF and iron-related indicators. Furthermore, a series of horizontal pleiotropy analyses, heterogeneity tests, and sensitivity analyses further clarified the robustness of the findings of this study. These findings will help healthcare professionals gain deeper insights into the role of iron status in the pathogenesis of HF and provide new ideas for the prevention and treatment of HF.

Iron, as an essential trace element for the human body, has very important biological functions. It is not only a necessary trace nutrient for many biochemical processes such as energy metabolism, oxygen transport, and redox reactions, but also plays a crucial role in the survival of cells.^[20]^ It through hemoglobin, is involved in the transport and storage of oxygen. However, when iron overload occurs, it can induce ferroptosis in cells through the Fenton reaction. These functions of iron make it closely related to the pathogenesis of HF, which includes mitochondrial dysfunction, oxidative stress, myocardial fibrosis, and cardiomyocyte apoptosis, indicating that both iron deficiency and iron overload are closely associated with HF.^[2,21]^ In clinical practice, serum iron, ferritin, TIBC, and TSAT are commonly used indicators to evaluate iron status. Ferritin, a spherical protein with a molecular weight of 480 kDa, consists of 2 types of subunits: light chains and heavy chains, and is primarily responsible for storing and releasing iron ions. Its serum level is closely related to the total amount of iron stored in the body and plays a critical role in many biological processes, including DNA synthesis, oxidative stress responses, cellular iron homeostasis, and mitochondrial respiration.^[22]^ Therefore, abnormal changes in serum ferritin levels have become an important pathogenic factor in cardiovascular diseases. However, in recent years, the conclusions of studies on serum ferritin levels and cardiovascular diseases have been inconsistent. A prospective study evaluated the correlation between iron metabolism markers and the prognosis of HF, finding that ferritin concentration was not related to prognosis, while low TSAT and serum iron were associated with the risk of all-cause mortality in HF.^[18]^ A cross-sectional study found that elevated serum ferritin was independently associated with carotid intima-media thickness and plaques in women.^[23]^ Meanwhile, 2 other cross-sectional studies found no association between serum ferritin and carotid atherosclerosis.^[24,25]^ The relationship between iron status and cardiovascular diseases has been controversial. Hunnicutt et al found that total iron intake and serum iron concentration are negatively correlated with CVD prevalence, while heme iron intake is positively correlated with CVD prevalence.^[26]^ Sung et al found that elevated ferritin is associated with coronary artery calcification and suggests that increased ferritin is a marker of early coronary atherosclerosis.^[27]^ Galan et al confirmed that in middle-aged individuals, ferritin is positively correlated with total cholesterol, triglycerides, obesity, blood pressure, and other cardiovascular disease risk factors.^[28]^ Therefore, there may be a positive correlation between serum ferritin and cardiovascular diseases and their risk factors. Additionally, a prospective cohort study found that both low and high serum ferritin levels are associated with a higher risk of heart HF in the general population.^[29]^ The results of this study provide evidence for a causal relationship between higher genetically predicted ferritin levels and a lower risk of HF.

Although the exact mechanism by which serum ferritin levels are associated with the risk of heart failure is not clear, related studies have suggested that oxidative stress, inflammatory response, and cell death may be involved.^[30]^ Firstly, iron is an essential trace element that plays a crucial role in the transport of oxygen in the blood.^[31]^ Measurement of serum ferritin concentration is considered the best noninvasive indicator of body iron stores.^[32]^ Research has shown that the heart-specific deletion of the ferritin heavy chain BFth1 leads to iron dysregulation and increased oxidative stress in the heart, thereby increasing susceptibility to tissue damage induced by iron overload.^[2]^ Furthermore, reduced serum ferritin levels may be related to enhanced inflammatory responses. Patients with heart HF exhibit a systemic inflammatory state, where inflammatory factors can cause an increase in hepcidin levels. Hepcidin binds to membrane iron transport proteins on the cell surface, promoting their internalization and degradation, which inhibits the transport of intracellular iron into circulation, leading to decreased release of stored iron.^[18]^ Finally, ferritin plays a crucial role in maintaining cardiac iron homeostasis and normal heart function. Decreased serum ferritin levels can lead to iron death through various mechanisms, including the release of iron ions, increased oxidative stress, and inflammatory responses. Research has shown that the absence of ferritin H in cardiomyocytes increases the production of ROS, leading to cardiac injury and heightened susceptibility to iron overload-related iron death and HF.^[30]^

This study has the following advantages. First, it reveals the causal relationship between serum ferritin and the risk of heart failure from a genetic perspective using a 2-sample bidirectional MR method. Compared to traditional observational studies, the use of genetic instruments is less influenced by lifestyle or environmental factors, reducing the impact of confounding factors and reverse causation. Second, this study selected a large-sample size of GWAS summary data and employed various methods for MR analysis, along with sensitivity analyses, which not only improved statistical power but also ensured the robustness of the results. In addition, all GWAS data in this study were obtained from European populations, which reduces the risk of population stratification bias. Finally, since GWAS data can be accessed from publicly available biobanks, MR analysis is more cost-effective compared to randomized controlled trials. However, this study also has some limitations. First, all GWAS data came from European populations, and the conclusions may not be generalizable to other ethnicities or regions, necessitating further data collection and analysis to confirm the universality of the research results. Second, in this study, aside from serum ferritin, the other 3 indicators related to iron status did not show a significant causal association with the risk of heart failure, which may be related to the relatively small number of HF cases in the databases, thus requiring a larger sample size to validate the aforementioned associations. In addition, the results of this study mainly involve iron status within the normal range for the general population and cannot be used to infer the iron status of individuals with extreme abnormalities. At the same time, this study is limited by the database and cannot conduct stratified analysis based on potential influencing factors such as gender and age. Finally, the mechanisms and clinical manifestations of HF are diverse and complex, this study only explains the causal relationship from a genetic level, and the potential biological mechanisms of this association have not been fully elucidated. Therefore, careful interpretation is needed in the context of clinical multifactorial backgrounds, and further in-depth exploration is required in the future.

In summary, this study employed a 2-sample bidirectional MR method to infer a causal relationship between higher serum ferritin levels and lower HF risk from a genetic perspective, providing new insights for the prevention and treatment of HF. However, future research still needs to conduct large-scale randomized controlled trials to further validate and elucidate the underlying mechanisms.

## Acknowledgments

The authors thank the studies or consortiums referenced and included in the present analysis for providing public datasets.

## Author contributions

**Data curation:** Yaqi Hu, Tao He, Jianhe Liu, Weisong Wang.

**Formal analysis:** Yaqi Hu.

**Investigation:** Yaqi Hu, Tao He, Jianhe Liu.

**Methodology:** Yaqi Hu, Tao He, Jianhe Liu, Weisong Wang.

**Validation:** Tao He.

**Writing – original draft:** Yaqi Hu.

**Writing – review & editing:** Yaqi Hu, Tao He, Jianhe Liu, Weisong Wang.
